# Cannabinoid Receptor Interacting Protein 1a (CRIP1a): Function and Structure

**DOI:** 10.3390/molecules24203672

**Published:** 2019-10-12

**Authors:** William T. Booth, Noah B. Walker, W. Todd Lowther, Allyn C. Howlett

**Affiliations:** 1 Department of Biochemistry and Center for Structural Biology, Wake Forest School of Medicine, Medical Center Blvd., Winston-Salem, NC 27157, USA; wbooth@jcsu.edu (W.T.B.); tlowther@wakehealth.edu (W.T.L.); 2 Department of Physiology and Pharmacology, Wake Forest School of Medicine, Medical Center Blvd., Winston-Salem, NC 27157, USA; nbwalker@wakehealth.edu; 3 Center for Molecular Signaling, Wake Forest University, 1834 Wake Forest Road, Winston-Salem, NC 27109, USA; 4 Department of Physiology and Pharmacology, Center for Research on Substance Use and Addiction, Wake Forest School of Medicine, Medical Center Blvd., Winston-Salem, NC 27157, USA

**Keywords:** adenylyl cyclase, β-arrestin, computational chemistry, CP55940, cyclic adenosine 3′,5′monophosphate (cAMP), extracellular signal-regulated kinase (ERK), G proteins, G protein coupled receptor (GPCR), WIN55212-2

## Abstract

Cannabinoid receptor interacting protein 1a (CRIP1a) is an important CB_1_ cannabinoid receptor-associated protein, first identified from a yeast two-hybrid screen to modulate CB_1_-mediated N-type Ca^2+^ currents. In this paper we review studies of CRIP1a function and structure based upon in vitro experiments and computational chemistry, which elucidate the specific mechanisms for the interaction of CRIP1a with CB_1_ receptors. N18TG2 neuronal cells overexpressing or silencing CRIP1a highlighted the ability of CRIP1 to regulate cyclic adenosine 3′,5′monophosphate (cAMP) production and extracellular signal-regulated kinase (ERK1/2) phosphorylation. These studies indicated that CRIP1a attenuates the G protein signaling cascade through modulating which Gi/o subtypes interact with the CB_1_ receptor. CRIP1a also attenuates CB_1_ receptor internalization via β-arrestin, suggesting that CRIP1a competes for β-arrestin binding to the CB_1_ receptor. Predictions of CRIP1a secondary structure suggest that residues 34-110 are minimally necessary for association with key amino acids within the distal C-terminus of the CB_1_ receptor, as well as the mGlu_8a_ metabotropic glutamate receptor. These interactions are disrupted through phosphorylation of serines and threonines in these regions. Through investigations of the function and structure of CRIP1a, new pharmacotherapies based upon the CRIP-CB_1_ receptor interaction can be designed to treat diseases such as epilepsy, motor dysfunctions and schizophrenia.

## 1. Introduction

Cellular signaling via the CB_1_ cannabinoid receptor has been predicated upon the understanding that ∆^9^-tetrahydrocannabinol (THC) is the primary psychoactive compound in *Cannabis* (reviewed in [1]), and that ∆^9^-THC analogs serve as agonists to promote G protein activation. Signal transduction occurs via Gi/o-mediated inhibition of adenylyl cyclase for agonist analogs of ∆^9^-THC, such as CP55940, prototypical of a series of cannabimimetic analgesics developed by Pfizer (reviewed in [2]). WIN55212-2 and its analogs are amino-alkylindole agonists for the CB_1_ receptor [2]. The CB_1_ receptor is a G protein-coupled receptor (GPCR) for the endogenous eicosanoids N-arachidonylethanolamide and 2-arachidonoylglycerol, and some structurally homologous lipid mediators (reviewed in [3,4]). In addition to Gi/o-mediated inhibition of adenylyl cyclase, CB_1_ agonists also release Gβγ subunits to inhibit various Ca^2+^ channels. CB_1_ agonists also promote complex interactions with receptor tyrosine kinases and non-receptor src kinases to activate the extracellular signal-regulated kinase (ERK1/2) and other regulatory kinases [2,5]. In addition to G proteins, the CB_1_ receptor interacts with β-arrestins, which serve as regulators of cellular signaling or receptor trafficking [6,7]. 

Cannabinoid receptor interacting protein 1a (CRIP1a) was sought and discovered as a result of findings by the Deborah Lewis laboratory that deletion of the distal C-terminal amino acids augmented the CB_1_ receptor constitutive inhibition of the voltage-dependent Ca^2+^ current in superior cervical ganglion neurons, suggesting that the distal C-terminal domain performed an inhibitory function [8,9]. Reasoning that the C-terminal binds to a regulatory protein, the Lewis laboratory performed a yeast two-hybrid screen which identified CRIP1a as a key CB_1_ receptor-associated protein [9,10]. They demonstrated that heterologous expression of CRIP1a could attenuate constitutive inhibition of N-type Ca^2+^ channels by exogenously expressed CB_1_ receptors in superior cervical ganglion neurons [10]. This finding suggests that Gβγ release can be attenuated in the presence of CRIP1a. Co-immunoprecipitation of CRIP1a with CB_1_ receptors demonstrated that these two proteins form a complex in 3-[(3-cholamidopropyl)dimethylammonio]-1-propanesulfonate (CHAPS) detergent-solubilized rat brain membranes [10]. Deletion mapping studies demonstrated that the distal C-terminal nine amino acids of the CB_1_ receptor constituted the minimum domain required for CRIP1a binding [10]. 

The purpose of the present review is to describe the unique interactions that CRIP1a can have with the CB_1_ receptor to regulate cellular signaling and CB_1_ receptor trafficking. Regulation of CB_1_ receptor activity by CRIP1a can be investigated at both the functional and structural levels with the goal of designing peptide or small molecule drugs that can selectively target the CRIP1a-CB_1_ receptor interaction for therapeutic intervention in the treatment of pain, convulsions [11,12,13,14], schizophrenia [15,16], and neurodegenerative motor disorders [17].

## 2. Functional Modulation of CB_1_ Cellular Signaling by CRIP1a 

Our laboratory has performed studies with N18TG2 neuroblastoma cell clones that endogenously express CB_1_ receptors, CRIP1a, G proteins, and other associated proteins as a model to investigate neuronal cell signaling and to provide insight into the interactions of these proteins in their native environment [18,19,20]. Using stable CRIP1a-overexpressing and CRIP1a-siRNA-silenced knockdown clones, we can manipulate these proteins in CRIP1a gene dose experiments to determine how these incremental increases or decreases in CRIP1a modulate the endogenous system. This model is superior to use of non-neuronal host cells (e.g., COS, HEK, CHO) that do not exhibit a neuronal phenotype and fail to express some or all of the native system components. 

### 2.1. G Protein Selectivity and Effect on Signal Transduction Via cAMP Inhibition and ERK1/2 Phosphorylation 

Studies in cell culture models have demonstrated that CRIP1a over-expression attenuates CB_1_ receptor signaling via G proteins [18,21]. As quantitated by antibody-capture scintillation proximity [^35^S]guanosine 5′-O-[gamma-thio]triphosphate (GTPγS) binding assays, CRIP1a influences the subtype of Gi/o protein that can be activated by agonists [18]. Over-expression of CRIP1a attenuated CP55940-activation of Gi3 or Go, whereas depletion of CRIP1a significantly increased activation of these G proteins. Concurrently, over-expression of CRIP1a led to a robust activation of Gi1 or Gi2 in response to CP55940. Both CP55940-mediated inhibition of cAMP accumulation as well as CP55940-driven ERK phosphorylation were accentuated by CRIP1a depletion [18]. At the level of cAMP regulation, the depletion of CRIP1a resulted in a left-shift of the log dose-response curve for CP55940, allowing the inhibition of cAMP to occur at much lower concentrations of agonist [18]. Initial (phase I) phosphorylation of ERK is governed by cAMP levels, as well as by release of βγ subunits from Gi/o proteins and interaction with integrins and receptor tyrosine kinases [22]. As endogenous levels of CRIP1a curtail agonist-mediated inhibition of cAMP production, the increased protein kinase A (PKA) activity leads to inactive src or raf, and thereby reduced ERK phosphorylation. When endogenous CRIP1a levels are depleted, cAMP levels and PKA activity are reduced, and ERK phosphorylation is increased, consistent with the data observed [18].

**Figure 1:**
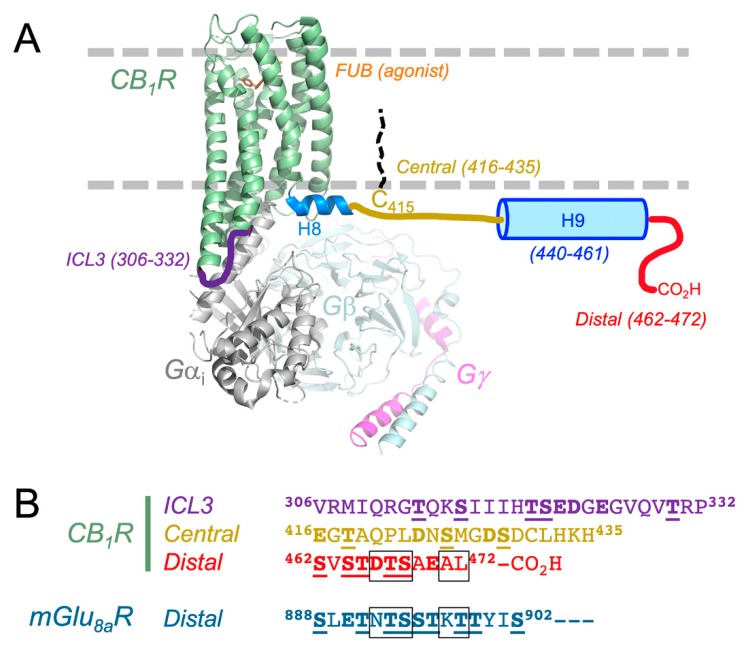
(**A**). Overview of the known structural and biochemical features of the CB_1_ receptor. The complex between full-length CB_1_ receptor, agonist MDMB-fubinaca (FUB), and G protein complex, as determined by cryoelectron microscopy (PDB code 6N4B) [32]. The intracellular region interactions are highlighted. Importantly, similar to available crystal structures, no electron density was observed for ICL3 and the C-terminus (residues 412–472). The nuclear magnetic resonance study by Ahn and colleagues supports that residues 440–461 form a second amphipathic helix that can interact with the membrane [33]. Cys415 can be palmitoylated to help anchor the C-terminus to the membrane [34,35]. (**B**). Primary sequence of the human CB_1_ receptor ICL3 loop, and C-terminal central and distal regions. The sequence is depicted in the same coloring as Figure 1A. Residues that can be phosphorylated are indicated in bold and underlined. Negatively-charged amino acids are indicated in bold. Five residues in the distal regions of the CB_1_ and mGlu_8a_ receptors important for CRIP1a binding are boxed in black (see text for details).

The CB_1_ receptor exists as a functional complex with Gi/o proteins in CHAPS detergent, which theoretically extracts lipid raft in addition to plasma membrane proteins [23]. This complex can be dissociated with non-hydrolyzable GTP analogs such as GTPγS, and by CB_1_ agonists with a significant degree of agonist selectivity [24,25]. In immunoprecipitations of the CB_1_-G protein complex from brain or N18TG2 membranes, peptides mimicking the juxtamembrane C-terminal of the CB_1_ receptor could compete for binding to Gαi3 and Gαo proteins. The competing peptides encompass the Helix 8 and the Cys415 palmitoylation site (Figure 1), suggesting that this domain is important for Gi3 and Go activation [25,26,27]. The juxtamembrane C-terminal peptide can function as an activator of Gi/o proteins in intact cells as a result of its conformation as an amphipathic helix which facilitates membrane crossing [28,29]. In contrast, competition by peptides from the intracellular loop 3 (ICL3) indicated that the CB_1_ receptor interaction with Gαi1 and Gαi2 involved the ICL3 but not the juxtamembrane C-terminal [30]. The direct association of CB_1_ receptor-Gi/o protein subtypes were subject to differential regulation by structurally different classes of agonists [24,30,31], suggesting a means of regulating efficacy by the CB_1_ receptor selection of its G protein partner. 

### 2.2. β-Arrestins and Internalization of the CB_1_ Receptor 

CRIP1a functions to diminish cell surface CB_1_ receptor density without altering total CB_1_ protein expression in N18TG2 neuroblastoma cells [18]. CRIP1a plays a role in CB_1_ receptor plasma membrane trafficking. CP55940 or WIN55212-2 reduced cell surface CB_1_ receptors via a dynamin- and clathrin-dependent internalization process [19]. CRIP1a over-expression attenuated the agonist-induced internalization of cell surface CB_1_ receptors, agonist-induced aggregation of transiently-expressed green fluorescent protein-CB_1_ receptors, and CP55940-mediated β-arrestin recruitment to punctae aggregates. Conversely, CRIP1a knock-down augmented agonist-mediated β-arrestin redistribution to punctae [20]. CP55940-mediated internalization of CB_1_ receptors was followed by a cycloheximide-sensitive recovery of surface receptors (30–120 min), suggesting the requirement for new protein synthesis. In contrast, WIN55212-2-mediated loss of cell surface CB_1_ receptors recovered only in CRIP1a-knockdown cells. These findings suggest an agonist bias in CRIP1a regulation of CB_1_ receptor trafficking from Golgi-endoplasmic reticulum to the plasma membrane.

Co-immunoprecipitation studies indicated that CB_1_ receptors associate in complexes with either CRIP1a or β-arrestin [20]. However, CRIP1a and β-arrestin fail to co-immunoprecipitate with each other. This suggests a competition by CRIP1a and β-arrestin for binding to the CB_1_ receptor, which could explain how CRIP1a attenuates the action of β-arrestin to mediate CB_1_ receptor internalization. 

## 3. Structure of CB_1_ Receptor and CRIP1a Interactions 

### 3.1. CB_1_ Receptor Structure: What is Known About How CRIP1a Interacts?

Several recent structural studies have dramatically improved our understanding of the CB_1_ receptor (see comprehensive review [36]). Two antagonist/inverse agonist crystal structures (taranabant, AM6538) revealed the first look at the protein in an inactive conformation [37,38]. The canonical 7-helical bundle is flanked perpendicularly by the amphipathic Helix 8 (Figure 1). The ligands bind within the orthosteric pocket, similar to the complexes of other class A GPCRs. The agonist AM11542 was developed and enabled the capture of the active conformation of the CB_1_ receptor [39]. The latter study identified a number of positional changes of many of the transmembrane helices and their associated extracellular and intracellular loops, resulting in a ~53% reduction in the volume of the ligand binding cavity. One important caveat of these studies is that the N- and C-termini of the CB_1_ receptor were truncated, along with many other site-directed sequence changes and loop alterations. Nonetheless, the structural changes observed in the CB_1_ receptor give insight into the propagation of ligand binding to G protein interactions and downstream signaling events.

Cryoelectron microscopy has subsequently been used to determine the complex between the full-length CB_1_ receptor, the synthetic agonist MDMB-fubinaca, and the Gi1 trimeric signaling complex (Figure 1) [32]. The positive allosteric modulator ZCZ-011 and the single-chain variable fragment of the monoclonal antibody scFv16 were used to further stabilize the complex for analysis. This study provides a rationale for the mechanism of G protein coupling and activation. Differences between the way that the ICL2 interacts with the Ras domain of Gαi1, when compared to other available nucleotide-free GPCR-G protein complexes, support the promiscuity of CB_1_ receptors for Gα_s_ and Gα_i_. Importantly, despite using the full-length CB_1_ receptor protein, the C-terminus was not visible in this structure.

With the use of circular dichroism and nuclear magnetic resonance spectroscopy, Ahn and colleagues [33] determined that the C-terminus of the CB_1_ receptor (residues 400–472, human protein numbering) contains two amphipathic helices when present in dodecylphosphocholine micelles as a membrane mimetic. As shown in Figure 1, Helix 8 (H8) is present as a highly conserved motif in rhodopsin-like GPCRs and is observable in the crystal structures of the CB_1_ receptor that are currently deposited in the protein data bank [37,38]. Helix 9 is located between the central and distal regions [33]. These regions contain multiple Ser and Thr phosphorylation sites (Figure 1), recently described as a “bar code” [40]. 

The CB_1_ receptor C-terminal region is critical for regulation of desensitization and receptor internalization in neurons and in cell models [41,42,43,44,45]. The CB_1_ receptor C-terminus possesses two separate domains functioning in signal desensitization versus receptor internalization [46,47,48,49], depicted in Figure 1A, with sequences in Figure 1B. Both domains possess Ser and Thr phosphorylation sites [20,50,51]. CB_1_ receptor-mediated desensitization of G protein-regulated potassium channel Kir3 [47] and glutamatergic neurotransmission [52] requires the CB_1_ receptor central C-terminal Ser426 and Ser430 targets of phosphorylation, and can be mediated by G protein receptor kinase 3 (GRK3) and β-arrestin2 [47,48]. Agonist-driven CB_1_ receptor internalization requires phosphorylation of the distal C-terminal patch of up to six Ser and Thr residues, recruitment of β-arrestin2, and mediation by clathrin and AP-2 [41,48]. In the N18TG2 cell model, phosphorylated central peptide competed for association with β-arrestin1; and phosphorylated central or distal peptides competed for association with β-arrestin2 [20].

Co-immunoprecipitation of CB_1_ receptor protein complexes demonstrated that either central or distal C-terminal peptides competed for the CB_1_ receptor association with CRIP1a [20]. These studies clearly demonstrate that phosphorylation of these sites determines whether CRIP1a or β-arrestins can associate with the CB_1_ receptor [19,20]. CRIP1a binds to the same regions of the receptor when in the unphosphorylated state [19,20]. It is also possible that residues in ICL3 may play a role in CRIP1a binding. 

In addition to the need for the unphosphorylated form of the CB_1_ receptor for CRIP1a binding, a few studies have highlighted the importance of key residues within the C-terminus of CB_1_ receptor. In the original yeast two-hybrid study that identified CRIP1a and its splice variant CRIP1b, the last nine amino acids of the CB_1_ receptor were required to maintain the interaction [10]. This study also tested a variety of truncation variants of CRIP1 and found that residues 34–110 could still bind to the CB_1_ receptor when expressed in yeast. A more recent study by Mascia and colleagues showed that full-length CRIP1a (residues 1–164) and the 1–110 and 34–110 truncation variants could block internalization of CB_1_ receptors expressed in HEK293 cells [53]. These observations are consistent with CRIP1a blocking β-arrestin2 binding [20]. Moreover, a panel of C-terminal truncation and alanine scanning variants of the CB_1_ receptor confirmed that the last nine amino acids, ^464^ST**DTS**AE**AL**^472^ (Figure 1B) were required for CRIP1a to prevent internalization [53]. The Ala-scan results, coupled with the testing of chimeras between the CB_1_ receptor and the mGlu_8a_ metabotropic glutamate receptor, demonstrated that five residues within the distal region were the most important to block receptor internalization (see boxed residues in Figure 1). Interestingly, if the sequence for mGlu_8a_ (^889^**NTS**ST**KT**^898^) was substituted with that of mGlu_8b_ (^889^**NSK**SS**VD**^898^), CRIP1a was not able to block receptor internalization [53]. It is also intriguing to observe that CRIP1a was able to interact with the mGlu_8a_ receptor, despite this receptor having the apparent recognition sequence within the context of a more extended C-terminus. Better knowledge of the crystal structure of CRIP1a and its complex with the CB_1_ or mGlu_8a_ receptors in the future should provide significant clarity to these observations.

### 3.2. Predicted Structure of CRIP1a

**Figure 2:**
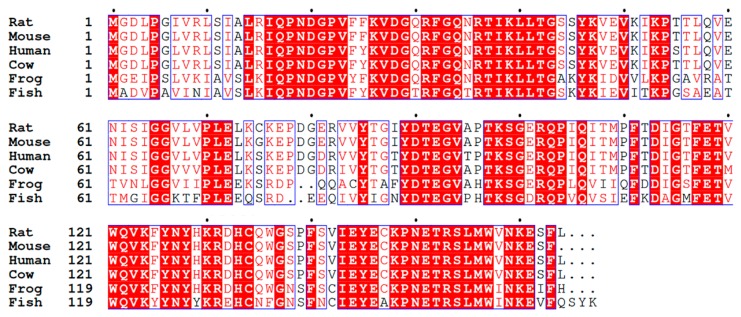
Primary sequence of CRIP1a from model organisms. Scientific names and protein codes: *Rattus norvegicus* (Q5M7A7), *Mus musculus* (Q5M8N0), *Homo sapiens* (Q96F85), *Bos taurus* (Q17QM9), *Xenopus laevis* (NP_001087998), *Danio rerio* (NP_001314699). The sequence identity relative to the top sequence proceeds down the alignment: 99%, 95%, 95%, 67%, and 59%.

The primary structure of CRIP1a has been identified and found to be highly conserved across species (Figure 2). However, there appears to be no amino acid sequence homology with other known proteins [10,54,55]. Due to the fact that there are currently no experimentally-determined structures of CRIP1a and CRIP1b, researchers have had to rely on ab initio modeling and in silico binding experiments in an attempt to develop and test hypotheses concerning the interaction between the CB_1_ receptor and CRIP1a/b. Computational studies on CRIP1a by Ahmed and colleagues [54] used a general strategy that included analysis of the protein sequence, and then used homology modeling to establish an ab initio model. The predicted model showed greatest structural homology with the protein guanine nucleotide dissociation inhibitor 2 (Rho-GDI2) [54,55]. The structure was energy minimized to satisfy chemical restraints and Ramachandran plot requirements. Rho-GDI2 structurally resembles a β-barrel protein, and is an important cell signaling molecule that regulates activation of the small G protein Rho [54]. It is important to remember, however, that given the low sequence identity (15.9 % and 25.7% sequence identity for CRIP1a and CRIP1b, respectively), the structure needs to be evaluated carefully. A subsequent study by Singh and colleagues confirmed the structure prediction for CRIP1a and extended the analysis to CRIP1b using similar methods [55].

Both groups then energy minimized the conformation of the CB_1_ distal C-terminal 9-mer peptide (residues 464–472) and docked the peptide onto the CRIP1a/b model surface. Multiple potential binding sites were identified and then ranked. Ahmed and colleagues proposed that the CRIP1a residues Asn61, Lys76, Arg82, Tyr85, Lys124, and Lys130 made direct hydrogen bonds to the 9-mer peptide [54]. In particular, they highlighted that the Lys130 side chain nitrogen atom stabilizes the backbone conformation of Ser464, Thr465, and Asp466. Similar studies of CRIP1b predicted that the binding pocket for the CB_1_ C-terminus included residues Asn61, Ile64, Leu68, Glu77, Asp79, Arg82, Tyr85, Tyr89, Cys126, Tyr124, and Met128 [55]. Given the ambiguity of the potential binding sites, significantly more work is required to understand the structure of CRIP1a/b and how it interacts with the CB_1_ receptor.

## 4. Conclusions

Our understanding of functional modulation of the CB_1_ receptor by CRIP1a has implicated CRIP1a as a competitor with certain Gi/o protein subtypes (Gi3, Go), as well as both β-arrestins1 and 2. The recently-identified interaction of the CB_1_ receptor with Gi1 can provide us with a greater understanding of how CRIP might function to promote CB_1_ receptor-Gi1 or Gi2 interactions. However, currently we still have a limited view of the totality of competitive interaction loci for Gi3, Go and the β-arrestins. Although the CRIP structure prediction results are promising, due to inconsistent evidence between the internalization assays and in silico analysis, continued efforts in structure determination by cryoelectron microscopy, x-ray crystallography, and/ or nuclear magnetic resonance could prove to be beneficial in providing a more detailed view of the CB_1_ receptor-CRIP1a interaction. A more accurate structure could provide greater insight and clarity into how the complete CB_1_ receptor intracellular surface could interact with CRIP1a.

CRIP1a has been linked to neuropathological states such as epilepsy and neurotoxicity in the hippocampus [11,12,13,14], schizophrenia phenotype [15,16], and motor dysfunction and addiction in the striatum [17], making the CRIP1a-CB_1_ receptor interaction an accessible target for novel drug design. This review focused on the structural interaction between the CB_1_ receptor and its association with CRIP1a. It is likely that this interaction will provide opportunities to develop either peptide “pepducins” or small molecule inhibitors specifically designed to modulate CB_1_ receptor function.
